# The facile synthesis of core–shell PtCu nanoparticles with superior electrocatalytic activity and stability in the hydrogen evolution reaction†

**DOI:** 10.1039/d1ra04001d

**Published:** 2021-08-02

**Authors:** Yongxiao Tuo, Qing Lu, Chen Chen, Tenglong Liu, Yuan Pan, Yan Zhou, Jun Zhang

**Affiliations:** School of Materials Science and Engineering, China University of Petroleum (East China) Qingdao 266580 China yxtuo@upc.edu.cn zhangj@upc.edu.cn; State Key Laboratory of Heavy Oil Processing, China University of Petroleum (East China) Qingdao 266580 China

## Abstract

Pt is the most efficient electrocatalyst for the hydrogen evolution reaction (HER); however, it is a high cost material with scarce resources. In order to balance performance and cost in a Pt-based electrocatalyst, we prepared a series of PtCu bimetallic nanoparticles (NPs) with different Pt/Cu ratios through a facile synthetic strategy to optimize the utilization of Pt atoms. PtCu NPs demonstrate a uniform particle size distribution with exposed (111) facets that are highly active for the HER. A synergetic effect between Pt and Cu leads to electron transfer from Pt to Cu, which is favorable for the desorption of H intermediates. Therefore, the as-synthesized carbon black (CB) supported PtCu catalysts showed enhanced catalytic performance in the HER compared with a commercial Pt/C electrocatalyst. Typically, Pt_1_Cu_3_/CB showed excellent HER performance, with only 10 mV (acid) and 17 mV (alkaline) overpotentials required to achieve a current density of 10 mA cm^−2^. This is because the Pt_1_Cu_3_ NPs, with a small average particle size (7.70 ± 0.04 nm) and Pt–Cu core and Pt-rich shell structure, display the highest electrochemically active surface area (24.7 m^2^ g_Pt_^−1^) out of the as-synthesized PtCu/CB samples. Furthermore, Pt_1_Cu_3_/CB showed good electrocatalytic stability, with current density drops of only 9.3% and 12.8% in acidic solution after 24 h and in alkaline solution after 9 h, respectively. This study may shed new light on the rational design of active and durable hydrogen evolution catalysts with low amounts of Pt.

## Abstract 2

A series of PtCu bimetallic nanoparticles with different Pt/Cu ratios was synthesized *via* a facile synthetic strategy to optimize the utilization of Pt atoms. Pt_1_Cu_3_/CB showed excellent HER performance under acidic and alkaline conditions.

## Graphical abstract

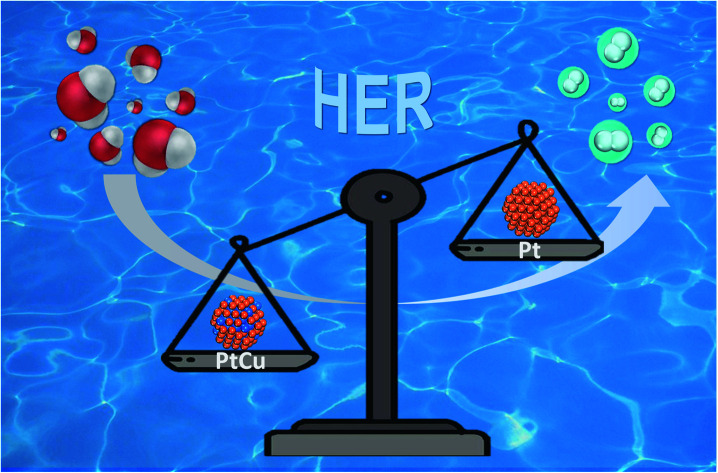

## 1. Introduction

Electrocatalysis has become one of the most efficient ways (after new energy sources, such as solar, wind, tidal, *etc.*) to generate renewable and green energy for preventing future energy crises and environment pollution.^1,2^ Of the electrochemistry-based conversion processes, electrocatalytic water splitting can provide useful oxygen and hydrogen gases with high purity.^3^ In particular, H_2_ is considered to be a clean reproducible energy carrier and important H donor in traditional industrial processes.^4–6^ Therefore, the half-reaction where protons and electrons are combined into molecular hydrogen, namely the hydrogen evolution reaction (HER), is now being widely investigated as a promising form of hydrogen production technology.^7–9^

After numerous studies, Pt-based catalysts are still the best choice compared with examples such as transition metal,^10–14^ metal phosphide (TMP),^15,16^ and metal nitride (TMN)^17,18^ catalysts. However, restricted by its expensive and scarce nature, the large-scale utilization of Pt-based electrocatalysts is impractical.^19^ Developing catalysts with high catalytic activity and durability, as well as low cost, remains a challenge relating to the hydrogen evolution reaction. To solve the above problems, many strategies have been adopted, which can be generally divided into two main approaches: (1) maximizing the efficiency of Pt *via* exposing all the Pt atoms or creating abundant defects;^20^ and (2) alloying Pt with other non-noble metals to reduce the dosage of Pt.^21^ Meanwhile, several comprehensive methods have been employed for the preparation of Pt-based electrocatalysts, including chemical vapor deposition (CVD),^22^ electrochemical deposition,^23^ and templating methods.^24^

Among the Pt metal alloys, the PtCu alloy is considered to be an ideal substitute due to its compatibility with Pt, high electrochemical stability, and the low cost of Cu.^25,26^ Researchers have synthesized some PtCu NPs with different geometrical structures *via* various preparation methods, such as nanocubes,^27^ nanospheres,^28^ and nanorods.^29^ According to previous studies,^25,30^ high electrocatalytic performance was shown by the (111) facet of Pt metal. The morphology of PtCu NPs can be significantly affected by the preparation method, including the Cu precursor species and nucleation differences between Pt and Cu, thus leading to the exposure of different crystal facets.^25,31–33^ Therefore, preparing PtCu NPs equipped with (111) crystalline planes is a good aim for the formation of high-efficiency Pt-based electrocatalysts. In addition, the utilization of carbon materials as Pt supports has been regarded as an efficient way to promote the HER performance due to the resulting high conductivity and large specific surface area.^34–36^

In this work, through a facile synthetic strategy, we prepared PtCu NPs with a uniform particle size distribution and exposed (111) facet. Then, carbon black was introduced to disperse the PtCu NPs and improve the conductivity of the material. The effects of the Pt/Cu ratio on the structural and chemical properties of the PtCu alloy were investigated and the relationship with the HER performance was studied. Due to the Pt–Cu core, Pt-rich shell structure, large electrochemically active surface area (24.7 m^2^ g_Pt_^−1^), and modified electronic structure of Pt, the as-synthesized Pt_1_Cu_3_/CB sample showed excellent HER electrocatalytic activity, with overpotentials of only 10 mV and 17 mV needed to achieve a current density of 10 mA cm^−2^ in acidic and alkaline electrolytes, respectively.

## 2. Experimental section

### 2.1 Materials

Oleylamine (Sigma-Aldrich, 70%), oleic acid (Sigma-Aldrich, 90%), Pt(acac)_2_ (Aladdin, 95%), CuCl (Sigma-Aldrich, 97%), W(CO)_6_ (Strem, 99% (<0.3% Mo)), Nafion (Sigma-Aldrich, 5 wt%), and *n*-butylamine (Sigma-Aldrich, 99%) were all used. Carbon black 350G was obtained from Keqing Corporation. The commercial Pt/C catalyst (10 wt% and 20 wt% loading) was obtained from Dalian Trico Chemical Co. Ltd. All reagents were of analytical grade and used without further purification.

### 2.2 Synthesis of PtCu NPs with different Pt/Cu ratios

PtCu NPs were synthesized using a modified high-temperature organic solution method.^25^ Briefly, 0.05 mmol of Pt(acac)_2_ and 0.05 mmol of CuCl were dissolved in a solution containing 9 mL of oleylamine and 1 mL of oleic acid, and this was heated to 140 °C under an argon flow. Then 0.14 mmol of W(CO)_6_ was quickly added into the system, and the reaction temperature was subsequently raised to 200 °C and kept at this level for 20 min under high-speed stirring. The resultant products were precipitated with ethanol followed by centrifugation. The obtained PtCu NPs were re-dispersed in hexane for the carbon-support process.

For convenience, the atomic ratio of Cu and Pt precursors was used to demonstrate the composition of the PtCu NPs. For example, Pt_1_Cu_3_ represents the nanoparticles synthesized with a 1 : 3 molar ratio of Pt precursor to Cu precursor.

### 2.3 Synthesis of PtCu/CB

Typically, to prepare the electrochemical catalyst with 20 wt% PtCu, 0.005 g of PtCu NPs dispersed in hexane was added into a mixture of 0.020 g of carbon black and hexane. The mixture was blended under ultrasonic conditions and stirred for 12 h. The product was centrifugated, washed with hexane several times, and dried overnight in a vacuum oven. Then the dried sample was re-dispersed into *n*-butylamine solution (99%), transferred into a 50 mL Teflon-lined stainless-steel autoclave, and maintained at 80 °C for 2 h to remove residual oleylamine. Finally, the PtCu/CB catalyst was washed with methanol to remove the remaining *n*-butylamine and dried at 65 °C in a vacuum oven overnight.

### 2.4 Characterization

Phase and crystal analysis of the samples was carried out *via* X-ray powder diffraction (XRD) studies, which were performed using a Philips X'pert pro MPD Super diffractometer equipped with Cu Kα radiation (*λ* = 1.5418 Å). The morphologies of the catalysts were analyzed using a Hitachi S-4800 field emission scanning electron microscope (SEM). The microstructures of the catalysts were characterized using high-resolution transmission electron microscopy (HRTEM, JEM-2100UHR) with an acceleration voltage of 200 kV. Energy dispersive spectroscopy (EDX) for mapping and cross-section compositional line profile analysis was carried out using FEI Tecnai G2 F20 S-Twin HRTEM apparatus at 200 kV. X-ray photoelectron spectroscopy (XPS) data were obtained using a Thermo Fisher ESCALAB 250 analyzer (Thermo Fisher Scientific, USA) with aluminum Kα radiation. Chemical component analysis was carried out *via* inductively coupled plasma-atomic emission spectrometry (ICP-OES) using an Agilent ICPOES 730 spectrometer. The Brunauer–Emmett–Teller (BET) surface areas of samples were measured with Micromeritics ASAP 2020 nitrogen adsorption apparatus *via* N_2_ physisorption at 77 K.

### 2.5 Electrochemical measurements

The electrochemical performances of samples were studied in a typical three-electrode system using a CHI 760E electrochemical workstation (Shanghai, China). A carbon-paper electrode coated with sample, Ag/AgCl, and carbon paper were employed as the working electrode, reference electrode, and counter electrode, respectively. During chronoamperometry testing, a Hg/HgO reference electrode was used to measure the alkaline electrolyte system. All reported potentials were converted to reversible hydrogen electrode (RHE) levels according to the following equation:

1 *E*_RHE_ = *E*_Ag/AgCl_ + 0.0591 × pH + 0.197

LSV measurements were carried out at a scan rate of 5 mV s^−1^ with 90% *iR*-compensation. Cyclic voltammetry (CV) experiments were conducted in 0.1 M aqueous HClO_4_ solution purged with N_2_ at a scan rate of 50 mV s^−1^. Before recording the CV profiles, the catalysts were activated *via* being scanned in 0.1 M HClO_4_ at 100 mV s^−1^ for 50 voltage cycles to obtain a clean surface. The hydrogen adsorption/desorption area was employed to estimate the electrochemically active surface area (EASA) as follows:2
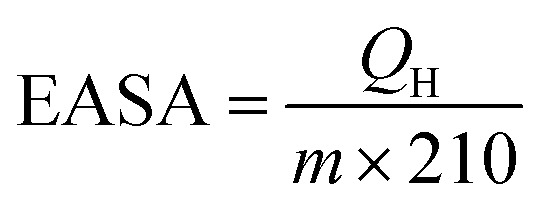
where *Q*_H_ is the charge for H desorption (μC), *m* represents the loading of Pt on the electrode (mg), and 210 is the charge required to oxidize a monolayer of H_2_ on Pt (μC cm^−2^). The Pt metal loading was calculated based on the ICP-OES results.

Typically, 5 mg of sample and 20 μL of Nafion solution (5 wt%, Dupont) were dispersed in 480 μL of ethanol under sonication for 30 min to form a homogeneous slurry. The 50 μL of slurry was loaded dropwise onto the carbon paper, which was then dried in air at room temperature. The electrolyte solution was purged with Ar for at least 30 min before each measurement.

## 3. Results and discussion

**Fig. 1:**
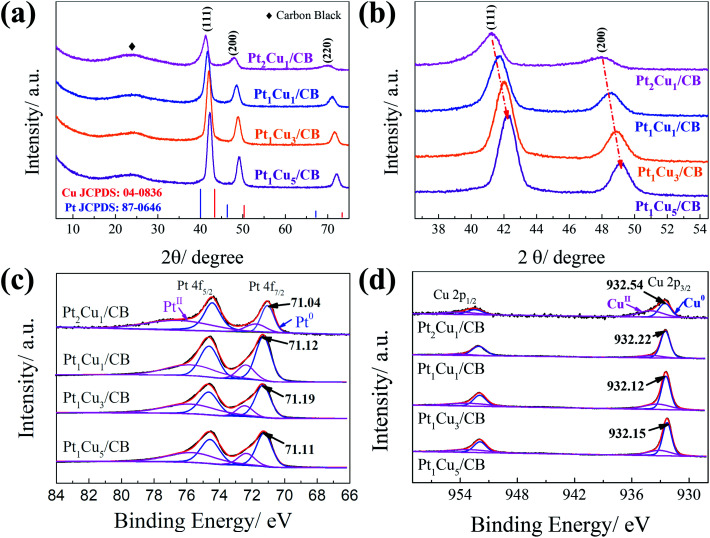
(a and b) XRD patterns of PtCu NPs with the reference patterns of Pt (JCPDS #87-0646) and Cu (JCPDS #04-0836). (c) Pt 4f and (d) Cu 2p high-resolution XPS spectra from PtCu/CB samples.

The PtCu NPs were prepared through the simultaneous reduction of Pt(acac)_2_ and CuCl at different Pt/Cu molar ratios using W(CO)_6_, and these were then uniformly dispersed onto carbon black to generate electrochemical catalysts.^37^ The XRD patterns of the as-synthesized samples are shown in Fig. 1(a), displaying typical peaks at 2*θ* values of 42.24°, 49.18°, and 72.12°, which are assigned to the (111), (200), and (220) planes, respectively, of fcc metal crystals. All the XRD patterns show that the peak positions of the PtCu NPs are between those of Pt (JCPDS #87-0646) and Cu (JCPDS #04-0836), indicating the formation of PtCu alloys. In addition, as shown in Fig. 1(b), the diffraction angles shift to higher values as the Cu ratio increases, which is consistent with a previous study.^38^ This is because the interplanar spacing of pure Cu is smaller than that of pure Pt; as the Cu ratio increases, the interplanar spacing of Pt–Cu will gradually become smaller. As revealed in previous studies,^39,40^ the high electrocatalytic performance could be ascribed to the (111) facets of the Pt-based alloys. As shown in Fig. 1(b), the as-synthesized PtCu NPs are dominated by the (111) facet; the increase in the intensity of the (220) peak with the Cu content suggests imperfect orientation along the 〈111〉 projection, confirming the existence of (111) textured arrays.^25^

XPS was used to study the surface compositions and chemical states of the as-synthesized PtCu/CB samples. As shown in Fig. S1,† the main peaks could be assigned to Pt 4f, C 1s, O 1s, and Cu 2p, confirming the existence of the corresponding elements. Fig. 1(c) and (d) shows the deconvolution of the high-resolution Pt 4f and Cu 2p spectra. The Pt 4f_7/2_ and Pt 4f_5/2_ peaks in the PtCu/CB samples shifted to higher binding energies compared with metallic Pt (71.0 and 74.5 eV) because of lattice compression in the bimetallic NPs (Table S3†). It should be noted that the binding energy of Pt shifts to a higher value as the Cu ratio increases, which could arise from electron transfer from Pt to Cu.^41^ This is also verified based on the downward shifting of the Cu peak binding energy (Fig. 1d). Interestingly, the Pt binding energy of Pt_1_Cu_5_/CB is slightly lower than that of Pt_1_Cu_3_/CB, indicating less electron transfer from Pt to Cu in the case of Pt_1_Cu_5_, which is also consistent with the Cu XPS spectrum, where the Cu binding energy of Pt_1_Cu_5_/CB is higher than that of Pt_1_Cu_3_/CB. This could be attributed to fewer interactions between Pt and Cu in Pt_1_Cu_5_/CB compared with Pt_1_Cu_3_/CB. It is well known that Pt_1_Cu_3_ has an ordered crystal structure, with Pt atoms located at face-centered planes and Cu atoms located at face-centered corners^38^. However, the dispersion of Pt and Cu in Pt_1_Cu_5_ may not be so uniform because of the unbalanced Pt/Cu ratio, thus causing insufficient interactions between Pt and Cu. The upward shift of the Pt 4f peaks was due to strong interactions between Cu and Pt, which would reduce the adsorption energies of reactive intermediates on the catalyst surface, thus improving the HER performance of PtCu/CB.^42–44^ In the Cu spectra, the peak intensities of metallic Cu are obviously larger than those of Cu(ii), revealing the metallic chemical state of Cu in the samples (Table S4†).^45^

**Fig. 2:**
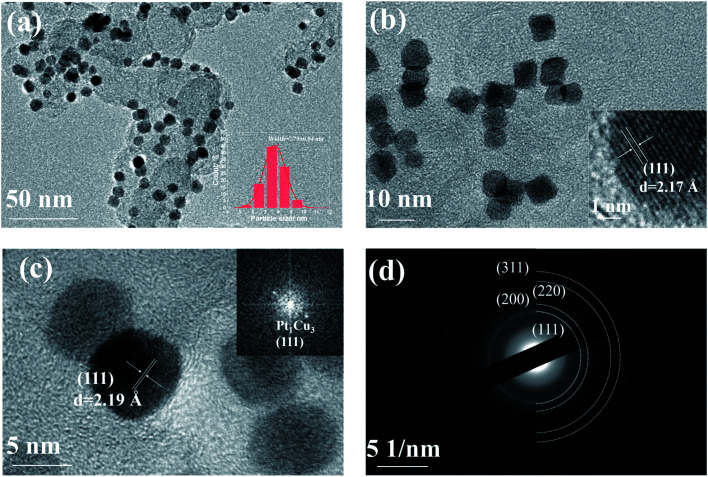
(a and b) TEM images, (c) a high-resolution TEM image, and (d) an SAED image of Pt_1_Cu_3_/CB. The insets in (a), (b), and (c) display the PtCu NP size distribution histogram, lattice spacing of Pt(111), and FFT pattern of Pt_1_Cu_3_/CB, respectively.

Based on TEM images (Fig. 2 and S2†), the PtCu NPs were seen to be uniformly dispersed on CB. As shown in Fig. 2(a), (b), S2(a), (d), and (g),† no obvious morphology changes can be observed upon altering the Pt/Cu ratio. The average diameters of the as-synthesized Pt_2_Cu_1_, Pt_1_Cu_1_, Pt_1_Cu_3_, and Pt_1_Cu_5_ NPs are 9.34 ± 0.28, 7.88 ± 0.02, 7.70 ± 0.04, and 8.45 ± 0.04 nm, respectively. This means that the Pt/Cu ratio affected the size of the PtCu alloy NPs. The high-resolution TEM images in Fig. 2(c), S2(b), (e), and (h)† demonstrate fringe spacing values of 0.227, 0.221, 0.219, and 0.219 nm for the (111) planes of Pt_2_Cu_1_, Pt_1_Cu_1_, Pt_1_Cu_3_, and Pt_1_Cu_5_, respectively. With an increase in the Cu precursor ratio, the fringe spacing of the (111) plane shrinks due to the replacement of Pt (0.225 nm) with Cu (0.208 nm); this matches well with the XRD results. The SAED image in Fig. 2(d) further confirms the formation of PtCu alloy NPs, with only one set of diffraction spots observed. The uniform distribution of PtCu NPs on CB was further confirmed based on SEM images (Fig. S3†), with no large NP aggregates observed. The pore structures of samples were investigated *via* N_2_ adsorption. All samples present a distinct type-IV curve with an obvious H1-type hysteresis loop in the *P*/*P*_0_ range from 0.42 to 0.95 (Fig. S4†), which is indicative of mesoporous characteristics.^46^ As shown in Table S2,† all the PtCu/CB hybrid catalysts showed a high BET surface area. The high specific surface area possessed by carbon black allows the enhanced dispersion of the PtCu NPs.

**Fig. 3:**
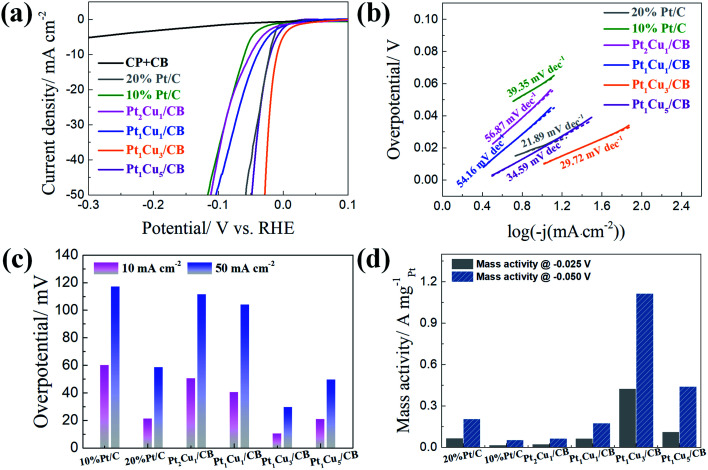
(a) HER polarization curves, (b) the corresponding Tafel plots, (c) the overpotentials at 10 mA cm^−2^ and 50 mA cm^−2^, and (d) the mass activities of Pt/C and PtCu/CB catalysts in 0.5 M H_2_SO_4_.

The electrocatalytic performances of the PtCu/CB catalysts with different Pt/Cu molar ratios in the HER were studied in Ar-saturated 0.5 M H_2_SO_4_ solution. As shown in Fig. 3(a) and (c), Pt_1_Cu_3_/CB displays the best HER catalytic activity out of the samples, with overpotentials of only 10 mV and 30 mV required to achieve current densities of 10 mA cm^−^ and 50 mA cm^−2^; these are also among the best values for previously reported Pt-based catalysts (Table S7†). The onset potential (*E*_onset_) of Pt_1_Cu_3_/CB (0 mV) is more positive than 10% Pt/C (−5 mV), Pt_2_Cu_1_/CB (−15 mV), Pt_1_Cu_1_/CB (−12 mV), and Pt_1_Cu_5_/CB (−10 mV). Tafel slopes were obtained from the polarization curves to investigate the inherent properties and reaction kinetics of the catalysts, as shown in Fig. 3(c). It is obvious that Pt_1_Cu_3_/CB has the second-lowest Tafel slope (29.72 mV dec^−1^), after that of the 20% Pt/C catalyst (21.89 mV dec^−1^), indicating its fast and efficient electron transfer kinetics compared to the other PtCu/CB samples (Table S5†).^47^ The Tafel slopes of the PtCu/CB samples vary from 29.72 to 56.87 mV dec^−1^ with different Pt/Cu ratios, indicating that the rate-determining recombination of ions and atom reaction processes on PtCu/CB follow the Volmer–Tafel reaction mechanism.^48,49^ The specific mass activity of Pt_1_Cu_3_/CB (1.115 A mg_Pt_^−1^ @ −0.05 V) is also the highest out of all the catalysts (Fig. 3(d)).

**Fig. 4:**
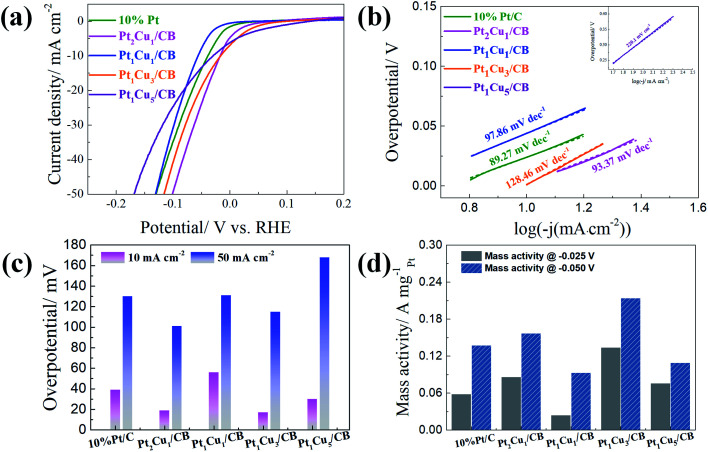
(a) HER polarization curves, (b) the corresponding Tafel plots, (c) the overpotentials at 10 mA cm^−2^ and 50 mA cm^−2^, and (d) the mass activities of Pt/C and PtCu/CB catalysts in 1.0 M KOH.

Fig. 4 displays the HER catalytic performances of PtCu/CB samples in 1.0 M KOH. Upon comparison (Table S6†), Pt_2_Cu_1_/CB shows the lowest Tafel slope (93.37 mV dec^−1^) and lowest overpotential (16 mV @ 10 mA cm^−2^ and 101 mV @ 50 mA cm^−2^) values. However, Pt_1_Cu_3_/CB has the highest specific mass activity value (0.214 A mg_Pt_^−1^) at −0.05 V, as shown in Fig. 4(d), larger than those of 10% Pt/C (0.138 A mg_Pt_^−1^), Pt_2_Cu_1_/CB (0.157 A mg_Pt_^−1^), Pt_1_Cu_1_/CB (0.093 A mg_Pt_^−1^), and Pt_1_Cu_5_/CB (0.110 A mg_Pt_^−1^). Therefore, Pt_1_Cu_3_/CB shows the best specific mass HER activity in alkaline solution, which is similar to its performance in 0.5 M H_2_SO_4_.

The resistance and charge transfer kinetics of an electrode material are important factors when fabricating high-performance HER electrocatalysts. Therefore, EIS studies were carried out to reveal the intrinsic resistance and charge transfer kinetics of PtCu/CB. As shown in Fig. S5(a),† the Nyquist plots of PtCu/CB samples show similar *R*_s_ values, indicating that PtCu/CB samples have similar intrinsic electron conductivity. However, the charge transfer kinetics are different, as shown in the inset of Fig. S5(a),† and Pt_1_Cu_3_/CB has the lowest *R*_ct_ value (0.18 Ω) compared to the other samples. In 1.0 M KOH, Pt_2_Cu_1_/CB shows the lowest *R*_ct_ value (5.41 Ω) compared to all the other samples, except for 10% Pt/C (Fig. S5(b)†), corresponding to its higher catalytic activity shown in Fig. 4(a).

**Fig. 5:**
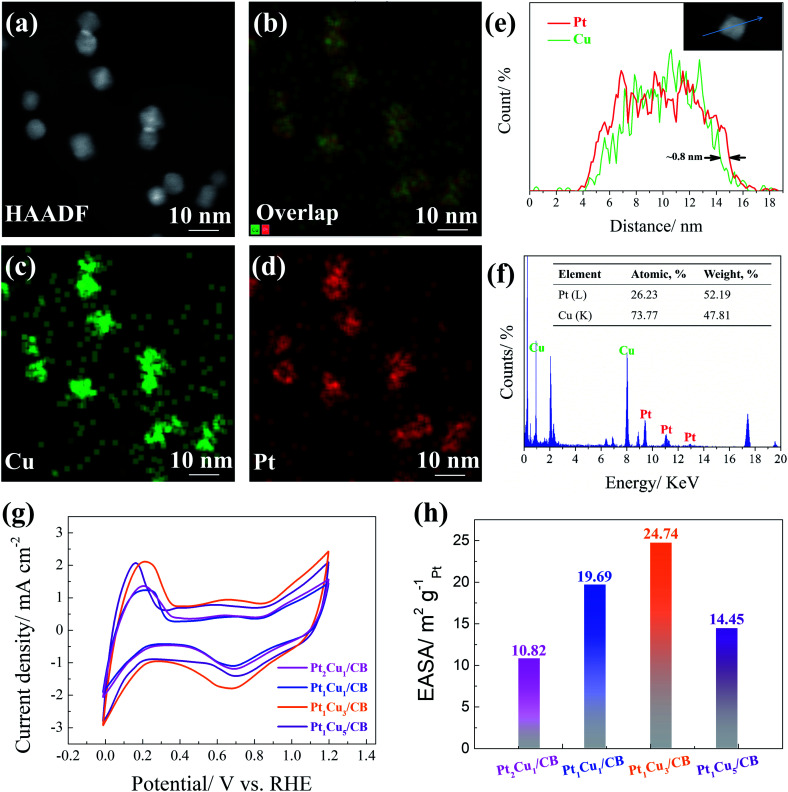
(a) A HAADF-STEM image, (b–d) EDX mapping images, (e) line-scanning profiles recorded along the path marked by the blue line shown in the inset, and (f) the EDS spectrum (the inset shows the weight and atomic ratios of Pt/Cu) for Pt_1_Cu_3_ NPs. (g) CV curves recorded in 0.1 M HClO_4_ solution purged with N_2_ at a sweep rate of 50 mV s^−1^ and (h) the EASA values of Pt_2_Cu_1_/CB, Pt_1_Cu_1_/CB, Pt_1_Cu_3_/CB, and Pt_1_Cu_5_/CB catalysts.

Based on the above observations, the relationship between the HER activity and Pt/Cu ratio follows a volcano trend, and the optimized ratio in this work is Pt_1_Cu_3_. To clarify the origin of the outstanding HER activity of Pt_1_Cu_3_/CB, the structural and chemical properties of the PtCu NPs were studied. Firstly, HAADF-STEM analysis and EDX analysis were used to investigate the distributions of different elements in the Pt_1_Cu_3_ NPs. The EDX mapping images in Fig. 5(a–d) illustrate the uniform distributions of Pt and Cu in the Pt_1_Cu_3_ NPs and the formation of the PtCu alloy. The line scanning profiles displayed in Fig. 5(e) confirm the Pt–Cu core and Pt-rich shell structure of the Pt_1_Cu_3_ NPs, and the thickness of the Pt shell is about 0.8 nm. This Pt-rich shell that is 3–4 atomic-layers thick significantly enhances the efficiency of Pt active sites. Fig. 5(f) shows the EDX pattern of Pt_1_Cu_3_/CB; the atomic ratio of Pt to Cu is about 26 : 74, which is consistent with the ICP-OES results (Table S1†). However, the surface Pt/Cu atomic ratio of Pt_1_Cu_3_/CB obtained *via* XPS is higher than this value (Table S1†), also revealing the surface segregation of Pt in the Pt_1_Cu_3_ NPs.

The electrochemically active surface area (EASA) results further verify the above discussion. Fig. 5(g) displays the cyclic voltammetry (CV) curves of the as-synthesized samples; the hydrogen adsorption/desorption region appears between 0.02 V and 0.4 V, and the oxidation/reduction peaks appear at about 0.4 V and span up to 0.9 V.^50,51^ As shown in Fig. 5(h), the EASA of the Pt_1_Cu_3_/CB sample is ∼24.74 m^2^ g_Pt_^−1^, which is the largest among those of Pt_2_Cu_1_/CB (10.82 m^2^ g_Pt_^−1^), Pt_1_Cu_1_/CB (14.45 m^2^ g_Pt_^−1^), and Pt_1_Cu_5_/CB (19.69 m^2^ g_Pt_^−1^). The Pt-rich shells of the Pt_1_Cu_3_ NPs should contribute to the large EASA of Pt_1_Cu_3_/CB, which can supply abundant active sites that are accessible to reactants.^52^

**Fig. 6:**
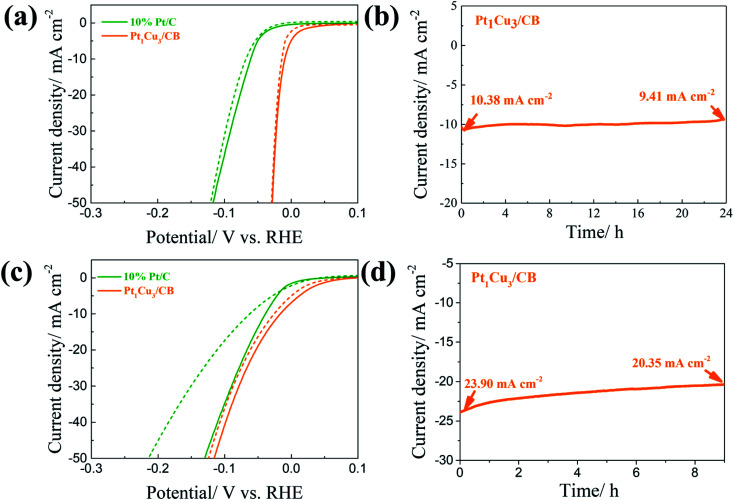
The HER polarization curves of commercial Pt/C and Pt_1_Cu_3_/CB catalysts before (solid lines) and after (dashed lines) 1000 cycles in (a) 0.5 M H_2_SO_4_ and (c) 1.0 M KOH. The chronoamperometric curves of the Pt_1_Cu_3_/CB catalyst in (b) 0.5 M H_2_SO_4_ at −0.083 V (*vs.* RHE) and in (d) 1.0 M KOH at −0.093 V (*vs.* RHE).

We also evaluated the stability of PtCu/CB catalysts *via* continuous potential cycling in acidic and alkaline solutions. As displayed in Fig. 6(a) and S6(a),† after 1000 cycles in 0.5 M H_2_SO_4_ between 0.1 and −0.153 V, the overpotentials at 50 mA cm^−2^ of 10% Pt/C, Pt_2_Cu_1_/CB, Pt_1_Cu_1_/CB, and Pt_1_Cu_5_/CB increased by 4, 32, 5, and 19 mV, respectively, whereas Pt_1_Cu_3_/CB shows almost no change after 1000 cycles. In 1.0 M KOH (Fig. 6(c) and S6(b)†), the overpotentials at 50 mA cm^−2^ of 10% Pt/C, Pt_2_Cu_1_/CB, Pt_1_Cu_1_/CB, and Pt_1_Cu_5_/CB increased by 85, 34, 20, and 32 mV after 1000 cycles, respectively. However, Pt_1_Cu_3_/CB shows excellent stability, as shown by the negligible change in overpotential (about 11 mV) in the alkaline electrolyte. Furthermore, the superior stability of Pt_1_Cu_3_/CB was confirmed *via* chronoamperometry measurements (Fig. 6(b) and (d)). At a constant voltage, the current density of the Pt_1_Cu_3_/CB electrocatalyst exhibits minor changes during the experiments (it dropped by 9.3% in acidic solution after 24 h and dropped by 12.8% in alkaline solution after 9 h), suggesting the good stability of Pt_1_Cu_3_/CB in both acidic and alkaline media. This could be ascribed to a synergistic effect between Pt and Cu, in which the Pt-rich shell prevents the leaching of Cu and the introduced Cu atoms prevent the oxidization of Pt. The amount of Cu lost from Pt_1_Cu_3_/CB was confirmed *via* analyzing the Cu content in the electrolyte after alkaline HER stability testing, and this is about 4.1%. Meanwhile the atomic ratio of post-HER Pt_1_Cu_3_/CB is the same as the pristine Pt_1_Cu_3_/CB catalyst (26 : 74). The morphology of Pt_1_Cu_3_/CB after the HER was also characterized *via* SEM. As displayed in Fig. S7,† it shows a similar morphology to the pristine catalyst (Fig. S3†). Therefore, the Pt_1_Cu_3_/CB catalyst is considerably stable during long-term electrocatalysis, without the leaching of Cu or Pt. Then, the chemical state of Pt in the Pt_1_Cu_3_/CB catalyst after stability testing was analyzed *via* XPS. As shown in Fig. S8,† the majority of surface Pt possess metallic phase characteristics, demonstrating only a slight difference from the pristine Pt_1_Cu_3_/CB catalyst. This indicates that a synergetic effect between Pt and Cu prevents the oxidation of Pt that is usually observed in Pt catalyst systems.^53^

Based on the above discussion, the remarkable catalytic performance of Pt_1_Cu_3_/CB in the HER could be ascribed to the following factors:^43^ (1) the high electrochemically active surface area of 24.7 m^2^ g_Pt_^−1^ due to the Pt–Cu core and Pt-rich shell structure; (2) the high (111) facet content, which is more favorable for the HER reaction; (3) electron transfer from Pt to Cu, which lowers the d-band center of Pt away from the Fermi level, facilitating the desorption of reaction intermediates; and (4) a synergetic effect between Pt and Cu, improving the stability of Pt through preventing the oxidization of Pt.

## 4. Conclusions

In summary, PtCu/CB catalysts synthesized *via* a facile method enhanced the utilization of Pt atoms and displayed excellent catalytic performance in the HER compared with a commercial Pt/C electrocatalyst. The PtCu NPs demonstrate a uniform particle size distribution with abundant (111) facets that are highly active for the HER. Electron transfer from Pt to Cu was observed in the PtCu NPs, which is favorable for the desorption of intermediates at Pt sites. Moreover, the Pt/Cu ratio in the PtCu NPs significantly affects the structural and chemical properties of the resulting PtCu alloys. Specifically, Pt_1_Cu_3_ NPs showed the minimum average particle size (7.70 ± 0.04 nm), with a Pt–Cu core and Pt-rich shell structure. Accordingly, the Pt_1_Cu_3_/CB electrocatalyst has the highest electrochemically active surface area (24.7 m^2^ g_Pt_^−1^) out of the as-synthesized PtCu/CB samples. Benefitting from these factors, the Pt_1_Cu_3_/CB electrocatalyst displayed excellent HER activity, with overpotentials of only 10 mV (acidic) and 17 mV (alkaline) needed to achieve a current density of 10 mA cm^−2^, and good stability, with a current density drop of 9.3% in 0.5 M H_2_SO_4_ after 24 h and a current density drop of 12.8% in 1.0 M KOH after 9 h.

## Author contributions

Yongxiao Tuo: methodology, investigation, writing; Qing Lu: validation, investigation; Chen Chen: software, formal analysis; Tenglong Liu: validation; Yuan Pan: review and editing; Yan Zhou: review and editing; Jun Zhang: supervision, funding acquisition.

## Conflicts of interest

The authors declare no conflicts of interest.

## Supplementary Material

RA-011-D1RA04001D-s001
