# Supplementary material for: The facile synthesis of core–shell PtCu nanoparticles with superior electrocatalytic activity and stability in the hydrogen evolution reaction
Source: RSC Adv. 2021 Aug 2;11(42):26326–35. doi: 10.1039/d1ra04001d (PMC9037382; doi:10.1039/d1ra04001d)
Supplement: RA-011-D1RA04001D-s001 [file RA-011-D1RA04001D-s001.pdf]

Electronic Supplementary Material (ESI) for RSC Advances.
This journal is © The Royal Society of Chemistry 2021

Facile Synthesis of Core-Shell PtCu Nanoparticles with Superior Electrocatalytic Activity and Stability in Hydrogen Evolution Reaction

Yongxiao Tuo[1, *], Qing Lu[2], Chen Chen[2], Tenglong Liu[2], Yuan Pan[2], Yan Zhou[1], Jun Zhang[1, 2,*]

1 School of Materials Science and Engineering, China University of Petroleum (East China), Qingdao, 266580, CHINA

2 State Key Laboratory of Heavy Oil Processing, China University of Petroleum (East China), Qingdao, 266580, CHINA

Corresponding Author:

Yongxiao Tuo: yxtuo@upc.edu.cn

Jun Zhang: zhangj@upc.edu.cn

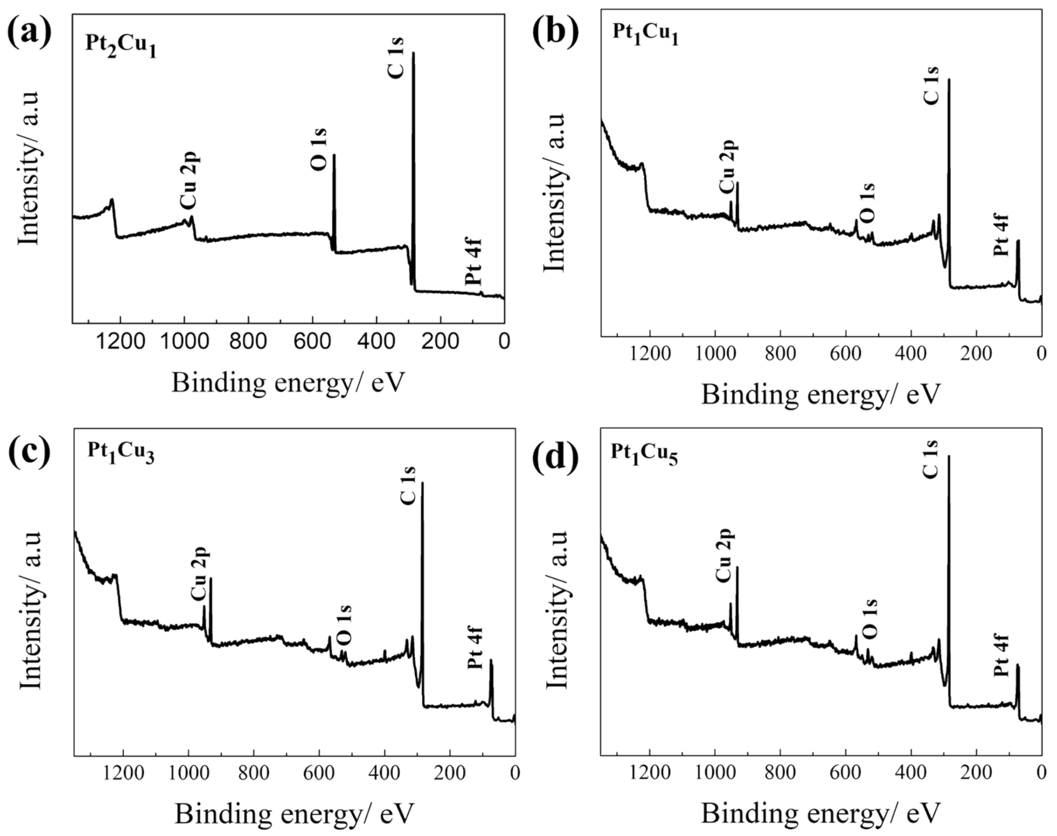

Figure S1 XPS wide-scan spectra of PtCu NPs.

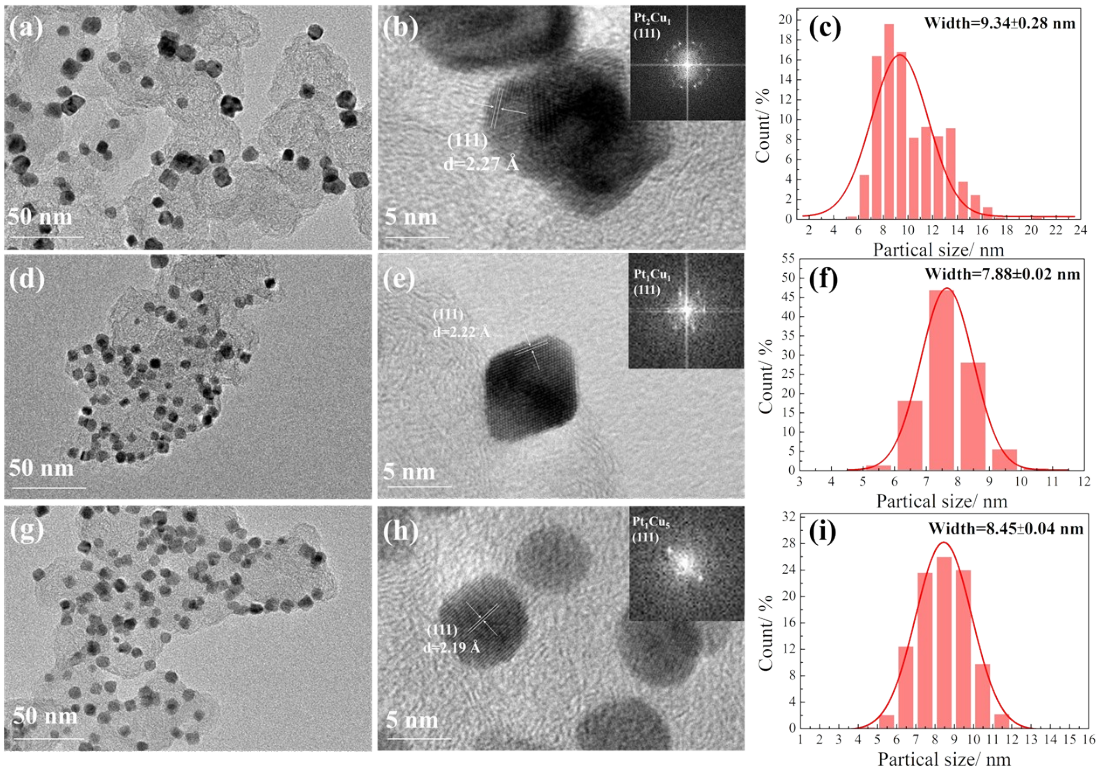

Figure S2 (a, d, g) TEM images, (b, e, h) HRTEM images and (c, f, i) particle size distribution histograms of PtCu NPs with different Pt/Cu ratio. The insertions in (b, e, h) display the FFT images of PtCu NPs.

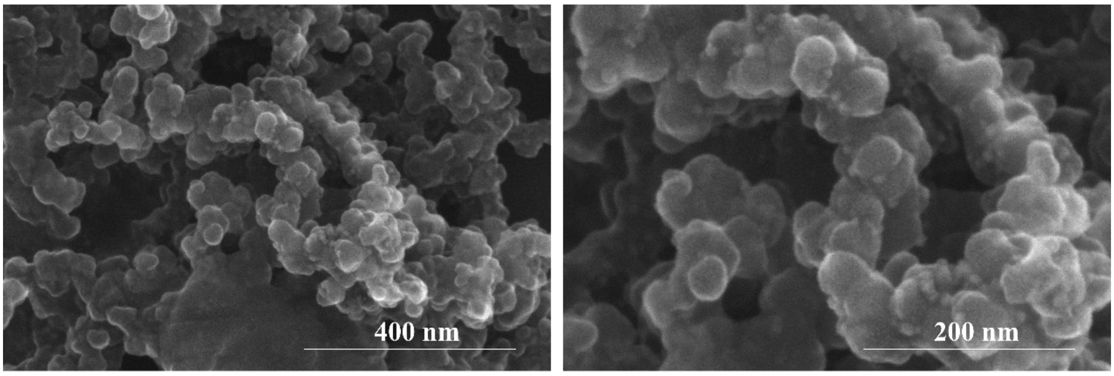

Figure S3 SEM images of Pt_1Cu_3/CB catalyst.

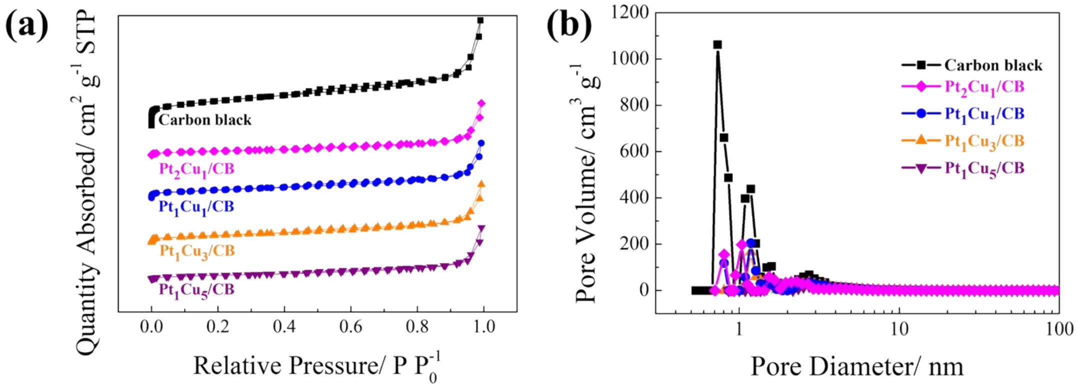

Figure S4 Nitrogen ad/desorption isotherms (a) and pore-size distribution curves (b) of carbon black and PtCu/CB samples.

Table S1 Pt/Cu mass ratio determined by ICP-OES and XPS.

Samples	ICP-OES	XPS
Pt_2Cu_1/CB	82.9:17.1	62.6:37.4
Pt_1Cu_1/CB	50.4:49.6	83.4:16.6
Pt_1Cu_3/CB	45.1:54.9	75.5:24.5
Pt_1Cu_5/CB	44.3:55.7	70.2:29.8

Table S2 Textural properties of as-synthesized PtCu/CB catalysts.

Samples	S_{BET}<br>$m^2 g^{-1}$	External surface<br>$m^2 g^{-1}$	BJH average pore size<br>nm	Total pore volume of pores<br>$cm^3 g^{-1}$
Carbon black	815.19	706.81	9.60	0.84
Pt_2Cu_1/CB	277.15	299.71	13.98	0.37
Pt_1Cu_1/CB	285.81	318.57	11.41	0.37
Pt_1Cu_3/CB	267.71	309.03	12.34	0.37
Pt_1Cu_5/CB	231.26	282.02	18.66	0.27

Table S3 XPS parameters of Pt 4f.

Sample	Pt $4f_{7/2}$				Pt $4f_{5/2}$			
	Pt^0		Pt^{II}		Pt^0		Pt^{II}	
	B.E., eV	Area, %	B.E., eV	Area, %	B.E., eV	Area, %	B.E., eV	Area, %
Pt_2Cu_1/CB	71.044	19.5	71.777	9.5	74.542	34.1	75.513	36.9
Pt_1Cu_1/CB	71.129	34.9	72.009	11.9	74.499	27.7	75.359	25.5
Pt_1Cu_3/CB	71.198	31.1	72.145	9.7	74.565	27.0	75.495	32.2
Pt_1Cu_5/CB	71.112	31.2	72.094	12.1	74.502	27.7	75.444	29.0

Table S4 XPS parameters of Cu 2p.

Sample	Cu $2p_{3/2}$				Cu $2p_{1/2}$			
	Cu^0		Cu^{II}		Cu^0		Cu^{II}	
	B.E., eV	Area, %	B.E., eV	Area, %	B.E., eV	Area, %	B.E., eV	Area, %
Pt_2Cu_1/CB	932.536	35.4	934.030	32.7	951.477	16.0	953.073	15.9
Pt_1Cu_1/CB	932.216	54.5	933.650	10.9	951.966	27.7	954.069	6.9
Pt_1Cu_3/CB	932.122	44.4	933.012	20.5	951.872	20.4	953.078	14.7
Pt_1Cu_5/CB	932.151	45.6	932.969	19.4	951.901	20.4	953.075	14.6

Table S5 Comparison of onset potential, overpotential, current densities, and Tafel slope value of various samples in 0.5M H_2SO_4.

Samples	E_{onset} ,mV	Tafel slope, mV dec^{-1}	η@j=10 mA·cm^{-2}, mV	η@j=50 mA cm^{-2}, mV
20% Pt/C	0	21.89	21.2	58.6
10% Pt/C	-5	36.80	60.1	117.1
Pt_2Cu_1/CB	-15	55.01	50.6	111.6
Pt_1Cu_1/CB	-12	51.56	40.5	104.1
Pt_1Cu_3/CB	0	26.28	10.5	29.7
Pt_1Cu_5/CB	-10	34.59	20.9	49.5

Table S6 Comparison of onset potential, overpotential, current densities, and Tafel slope value of various samples in 1.0M KOH.

Samples	E_{onset} ,mV	Tafel slope, mV dec^{-1}	η@j=10 mA·cm^{-2}, mV	η@j=50 mA cm^{-2}, mV
10% Pt/C	-2	45.76	39	130
Pt_2Cu_1/CB	-16	42.54	16	101
Pt_1Cu_1/CB	-27	42.73	56	131
Pt_1Cu_3/CB	-11	76.87	17	115
Pt_1Cu_5/CB	-10	139.49	30	168

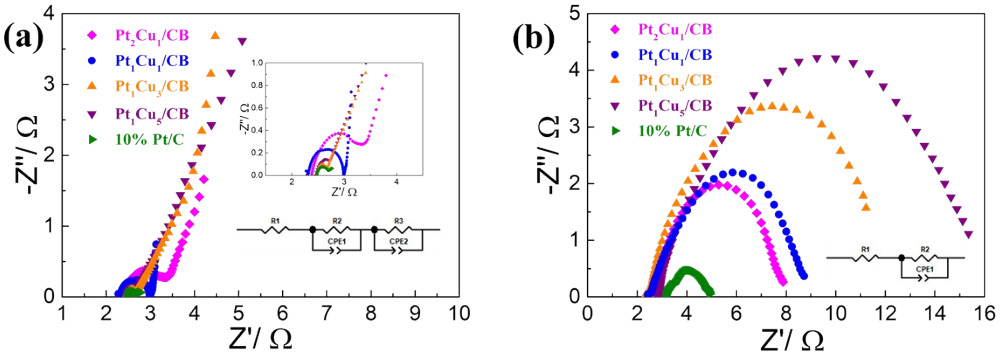

Figure S5 The EIS Nyquist plots of PtCu/CB and 10% Pt/C samples (a) in 0.5M H_2SO_4 at -0.223 V (vs. RHE) and (b) in 1.0M KOH at -0.010 V (vs. RHE).

Table S7 Comparison of HER performance of Pt-based catalysts in acid and alkaline electrolytes.

Electrolyte	Catalyst	Mass activity @-50 mV, A mg_{Pt}^{-1}	E_{onset}, mV	Tafel slope, mV dec^{-1}	η@j=10 mA·cm^{-2}, mV	Reference
H_2SO_4	Pt_1Cu_3/CB	1.1154	0	26.28	10	This work
	20% Pt/C	0.2067	0	21.89	21.2	This work
	10% Pt/C	0.0543	-5	36.80	60.1	This work
	PtCu DNFs	0.2523	0	34	27	[1]
	PtCu NPHs	0.0332	21	49	66	
	Pt CNFs	-	-	40	-	[2]
	PdCo@CN	-	-	31	80	[3]
	Cu-Pt		-0.01		j= -2.75 mA cm^{-2}@ -0.5 V	[4]
	Cu@Pt/4-Sulfophenyl/GC	28	-	-	-	[5]
	$Pt_1Cu_{1.03}$-D	19.34@-0.2V	-	28.5	20	[6]
	Pd@PtCu/C	-	-	26.2	19	[7]
	PtNi	-	-380	119	-	[8]
	$Pt_{24}Cu_{76}$ NFs	0.74@-0.9V	-	52	18	[9]
	Pt-WC-1,10/BMZ	6.659@-0.033V	-	40.1	23	[10]
KOH	Pt_1Cu_3/CB	0.2141	0	122.34	17	This work
	10 % Pt/C	0.1375	-5	87.70	39	This work
	PtCu NPHs	0.0470	-3	76	61	[1]
	PtCu DNFs	0.1503	0	55	36	
	PdCo@CN	-	-	-	250	[3]
	Cu@Ni	-	-	79	140	[11]

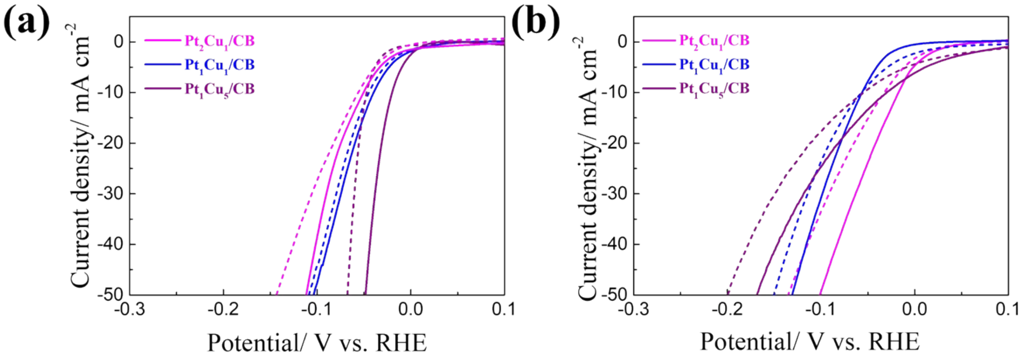

Figure S6 HER polarization curves of PtCu/CB catalysts before and after 1000 cycles in (a) 0.5M H_2SO_4 and in (c) 1.0M KOH.

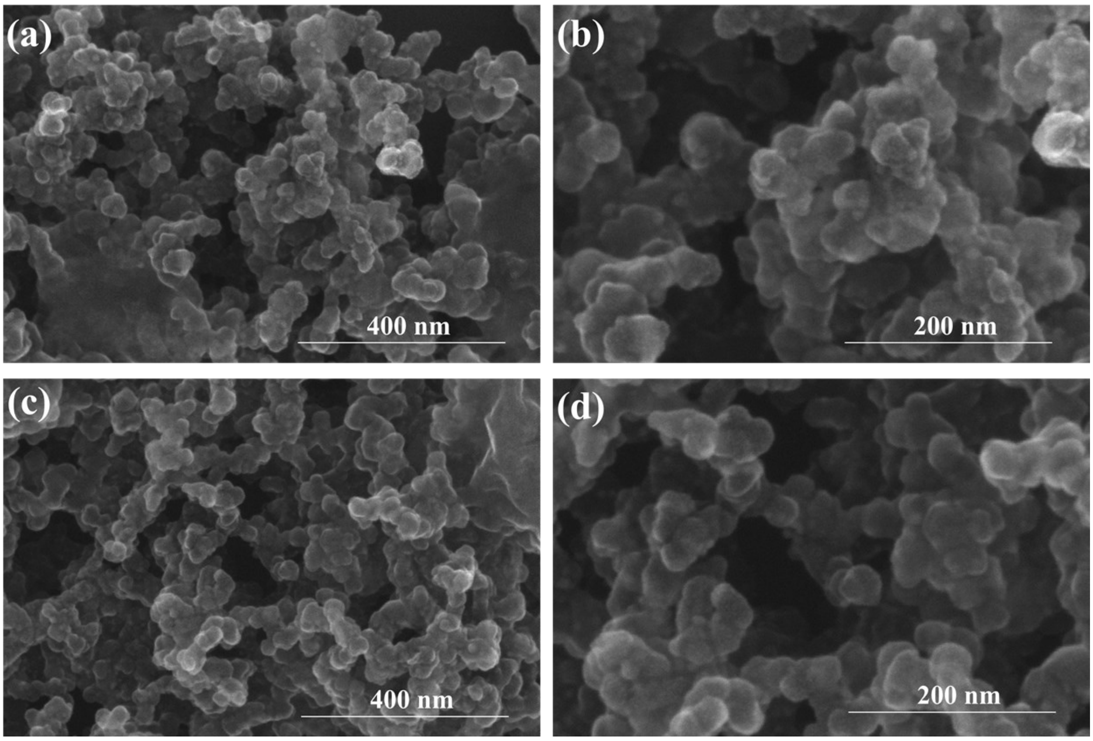

Figure S7 SEM images of Pt_1Cu_3/CB catalyst after (a, b) 9 h alkaline HER and (c, d) 24 h acidic HER stability test.

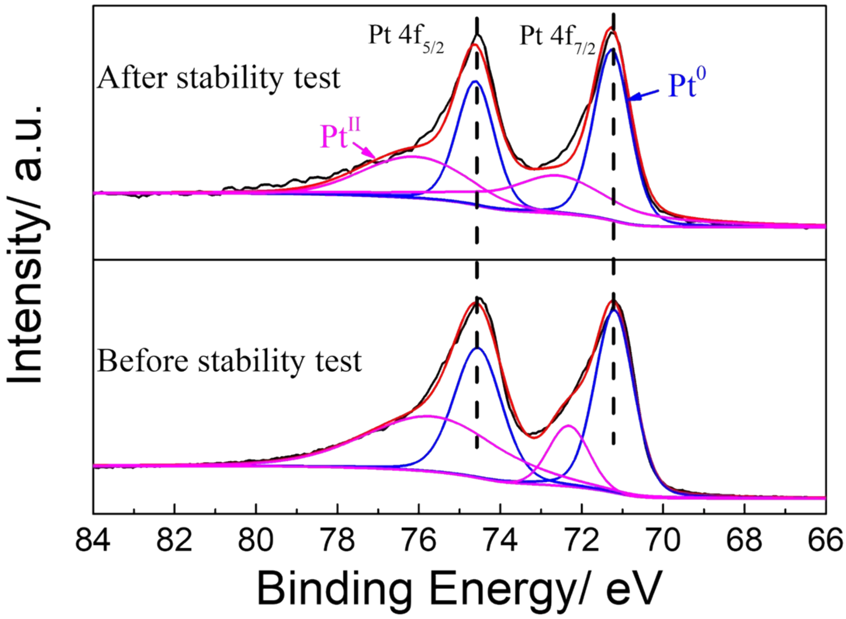

Figure S8 Comparison of Pt 4f XPS spectra between the as-prepared Pt_1Cu_3/CB catalyst and the corresponding one after 9 h alkaline HER stability test.

Reference

[1] Zhang, X. F., et al., *Solvothermal synthesis of monodisperse PtCu dodecahedral nanoframes with enhanced catalytic activity and durability for hydrogen evolution reaction.* ACS Applied Energy Materials, 2018. **1**(9): p. 5054-5061.

[2] Ghasemi, S., S.R. Hosseini, and S. Nabipour, *Preparation of nanohybrid electrocatalyst based on reduced graphene oxide sheets decorated with Pt nanoparticles for hydrogen evolution reaction.* Journal of the Iranian Chemical Society, 2019. **16**(1): p. 101-109.

[3] Chen, J., et al., *Active and durable hydrogen evolution reaction catalyst derived from Pd-doped metal–organic frameworks.* ACS applied materials & interfaces, 2016. **8**(21): p. 13378-13383.

[4] Mandegarzad, S., et al., *Cu-Pt bimetallic nanoparticles supported metal organic framework-derived nanoporous carbon as a catalyst for hydrogen evolution reaction.* Electrochimica Acta, 2016. **190**: p. 729-736.

[5] Mirzaei, P., et al., *Bimetallic Cu–Rh Nanoparticles on Diazonium-Modified Carbon Powders for the Electrocatalytic Reduction of Nitrates.* Langmuir, 2019. **35**(45): p. 14428-14436.

[6] Jia, Y., et al., *Sandwich-Type Electrochemical Immunosensor Based on Signal Amplification System of Porous PtCu Decorated FCuS Nanospheres for CEA Detection.* Journal of The Electrochemical Society, 2019. **166**(8): p. B713-B719.

[7] Bao, M., et al., *Surface evolution of PtCu alloy shell over Pd nanocrystals leads to superior hydrogen evolution and oxygen reduction reactions.* ACS Energy Letters, 2018. **3**(4): p. 940-945.

[8] Zheng, J., *Seawater splitting for high-efficiency hydrogen evolution by alloyed PtNix electrocatalysts.* Applied Surface Science, 2017. **413**: p. 360-365.

[9] Huang, X.-Y., et al., *L-proline assisted solvothermal preparation of Cu-rich rhombic dodecahedral PtCu nanoframes as advanced electrocatalysts for oxygen reduction and hydrogen evolution reactions.* Electrochimica Acta, 2019. **299**: p. 89-97.

[10] Chen, X., et al., *Coupling low platinum and tungsten carbide supported on ZIFs-Derived porous carbon for efficient hydrogen evolution.* Electrochimica Acta, 2019. **328**: p. 135077.

[11] Li, Z., et al., *Mesoporous Hollow Cu–Ni Alloy Nanocage from Core–Shell Cu@ Ni Nanocube for Efficient Hydrogen Evolution Reaction.* ACS Catalysis, 2019. **9**(6): p. 5084-5095.
